# The experience of linking Victorian emergency medical service trauma data

**DOI:** 10.1186/1472-6947-8-52

**Published:** 2008-11-17

**Authors:** Malcolm J Boyle

**Affiliations:** 1 Monash University, Department of Community Emergency Health and Paramedic Practice, P.O. Box 527, Frankston 3199, Victoria, Australia

## Abstract

**Background:**

The linking of a large Emergency Medical Service (EMS) dataset with the Victorian Department of Human Services (DHS) hospital datasets and Victorian State Trauma Outcome Registry and Monitoring (VSTORM) dataset to determine patient outcomes has not previously been undertaken in Victoria. The objective of this study was to identify the linkage rate of a large EMS trauma dataset with the Department of Human Services hospital datasets and VSTORM dataset.

**Methods:**

The linking of an EMS trauma dataset to the hospital datasets utilised deterministic and probabilistic matching. The linking of three EMS trauma datasets to the VSTORM dataset utilised deterministic, probabilistic and manual matching.

**Results:**

There were 66.7% of patients from the EMS dataset located in the VEMD. There were 96% of patients located in the VAED who were defined in the VEMD as being admitted to hospital. 3.7% of patients located in the VAED could not be found in the VEMD due to hospitals not reporting to the VEMD. For the EMS datasets, there was a 146% increase in successful links with the trauma profile dataset, a 221% increase in successful links with the mechanism of injury only dataset, and a 46% increase with sudden deterioration dataset, to VSTORM when using manual compared to deterministic matching.

**Conclusion:**

This study has demonstrated that EMS data can be successfully linked to other health related datasets using deterministic and probabilistic matching with varying levels of success. The quality of EMS data needs to be improved to ensure better linkage success rates with other health related datasets.

## Background

Record linkage can be used to combine different datasets about of person or medical event to allow for further indepth investigation. The combining, or linking, of two separate datasets was first proposed by Dunn in 1946 [1] with Newcombe and colleagues, in the late 1950' and 1960's, pioneering work on the linking of medical records. [2,3] In recent times data linking has been used to link large population datasets to further investigate health issues and trends in health and healthcare.

The use of EMS data for the purpose of linking one of more datasets in Australia dates back to the late 1980s when Ferrante et al linked Western Australian EMS data with hospital data during the process of developing the Road Injury Database. [4] Other international studies utilising EMS data in linkage studies have been undertaken to identify the error rate of data collection by emergency medical technicians compared to emergency department staff [5], to determine the quality of EMS and hospital emergency department data for patients who had been assaulted and transported to hospital [6], and to evaluate the performance of EMS [7].

Manual matching is seen as the "gold standard" in data linking, however this is often not feasible, if not impossible, due to the time consuming nature of the process, especially when using large population datasets. Deterministic matching using unique variables in two datasets is achievable in small datasets with accurate data. However, for large datasets probabilistic matching is the most efficient method. [8]

Only one previous prehospital data linking study from Australia has been published, this was a study using the Western Australia Data Linkage System and data from St John Ambulance Australia (WA Ambulance Service Incorporated) to investigate the outcomes of prehospital cardiac arrest patients.[9] The linking of Victorian Emergency Medical Service (EMS) data to Victorian Department of Human Services (DHS) hospital datasets has only been done previously with small test datasets, with no additional work undertaken to investigate patient outcomes. There is no published literature on the linking of Victorian EMS datasets to the Victorian State Trauma Outcome Registry and Monitoring (VSTORM) dataset.

The objective of this study was to identify the linkage rate of a large EMS trauma dataset with the Department of Human Services hospital datasets and the VSTORM dataset.

## Methods

**Figure 1:**
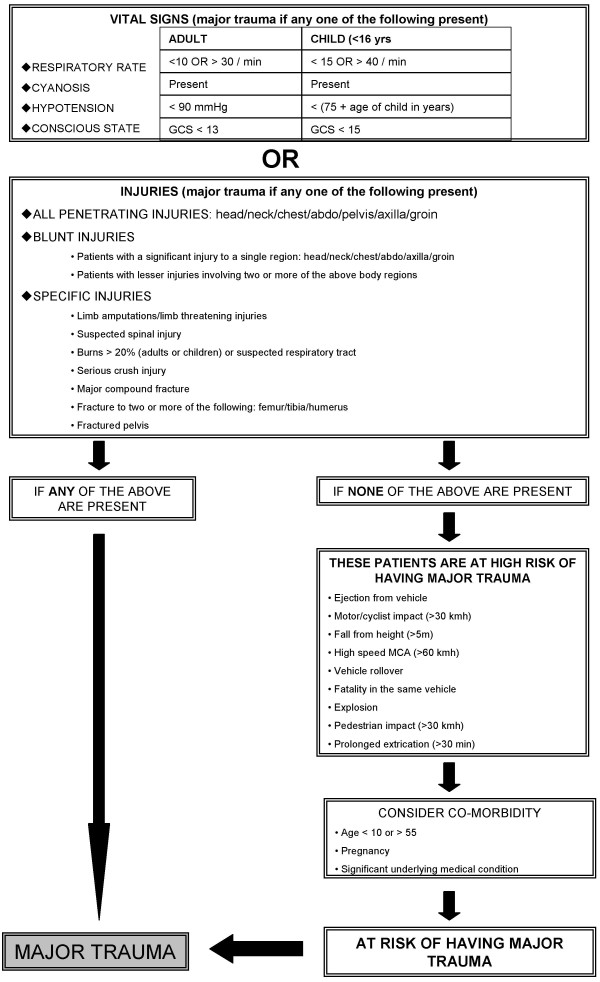
**Victorian prehospital major trauma criteria**. Copyright: Prehospital and Disaster Medicine.

This study reviews the linking of EMS trauma datasets to the Victorian DHS hospital datasets and the VSTORM dataset to determine which patients, who had sustained prehospital potential major trauma, as defined in figure 1, subsequently went on to have hospital defined major trauma, as listed in Table 1.

**Table 1:** Hospital Defined Major Trauma

• Death after injury
• Admission to an Intensive Care Unit for more than 24 hours, requiring mechanical ventilation
• Urgent surgery for intracranial, intrathoracic, or intraabdominal injury, or for fixation of pelvic or spinal fractures
• Injury Severity Score (ISS) > 15
• Serious injury to two or more body systems (excluding integumentary)

Trauma patients transported by emergency EMS in Victoria from the 1^st ^January 2002 to the 31^st ^December 2002 were eligible for inclusion in the study.

Data collected by the study was from Victoria, a south eastern state of Australia. Victoria covers approximately 227,590 square kilometres with a population of approximately 4.9 million people during the study period (49% males and 51% females). [10]

No electronic EMS clinical data repository was available in Victoria at the time of data collection. Consequently, each EMS Patient Care Record (PCR) for 2002 was manually reviewed. All trauma PCRs were retrieved, and then each individual trauma incident was analysed to establish eligibility for inclusion into the Victorian Prehospital Trauma Triage Study with PCR data entered into a secure database. The trauma definitions, EMS response, inclusion and exclusion criteria, and other data handling have been reported elsewhere.[11] Terms such as variable, deterministic matching, probabilistic matching, and manual matching are defined in Table 2.

**Table 2:** Definition of Terms

**Term**	**Definition**
Variable	An entity that can be used to store a value based on predefined criteria, e.g. the variable DOB stores a person's date of birth in a pre-defined date format, 99/99/9999
Deterministic matching	The electronic matching of one dataset to another using unique variables present in both datasets.
Probabilistic matching	The electronic matching of one dataset to another using similar or the same variables in both datasets, the matching is based on the probability (likelihood ratio theory) that the record in one dataset is the same as the record in another dataset.
Manual matching	The matching of one dataset to another by a person comparing each record in one dataset with each record in a second dataset.

Deterministic matching was undertaken using Microsoft Access™ (Microsoft Corporation, Version 10 SR2, Redmond, Washington, U.S.A.). Probabilistic matching was undertaken using Link Plus (Version 2.0, Centers for Disease Control and Prevention, Atlanta, Georga, U.S.A.).

As EMS in Victoria do not have individual ethics committees, ethics approval for the study was obtained from the Monash University Standing Committee for Ethics in Research on Humans and the Victorian Department of Human Services Ethics Committee.

## EMS Datasets

There were three EMS datasets created and used in the final linking process. The trauma profile dataset contained basic trauma data including, mechanism of injury, pattern of injury, and physiological status, and other data reported but not analysed, for example, standing falls, for all trauma patients. The trauma profile data has been reported elsewhere. [11] The trauma profile dataset contained 27,600 records.

The mechanism of injury only and sudden deterioration datasets were created as a part of sub-studies within the Victorian Prehospital Trauma Triage Study. These studies have been reported elsewhere. [12,13] The mechanism of injury only dataset contained 4,571 records and the sudden deterioration dataset contained 2,893 records (with 2,687 used for analysis).

Records from the mechanism of injury only and sudden deterioration datasets were also in the trauma profile dataset, however, there was less patient type data, e.g. patient observations. None of the datasets contained the patient name, address details, or other personal identifying type information, e.g. pension number, but the mechanism of injury only and sudden deterioration datasets did contain the patient's date of birth, the location and postcode of the trauma incident and the hospital they were transport to.

## Victorian Department of Human Services Datasets

In Victoria, the Department of Human Services (DHS) has two main hospital datasets, the first is the Victorian Emergency Minimum Dataset (VEMD), it contains data from emergency departments from approximately half of the state's registered public hospitals. Second, is the Victorian Admitted Episode Dataset (VAED), it contains hospital inpatient data from all state public hospitals and some private hospitals. DHS has predefined matching processes for linking the two hospital datasets.

## Victorian State Trauma Outcome Registry and Monitoring Dataset

The VSTORM dataset used in this study was a subset of the main dataset. The variables included date of incident, EMS case number, gender, date of birth, postcode of the incident location, destination hospital, EMS (Metropolitan Ambulance Service [MAS] or Rural Ambulance Victoria [RAV]) and hospital major trauma criteria (Table 1).

The VSTORM data were stored in a specifically written Microsoft Access™ database. The criteria for patients to be entered into VSTORM are listed in Table 1. There were 1,193 major trauma patients for 2002 identified in VSTORM, of these 1,096 (91.9%) were transported to hospital by EMS as verified by an EMS PCR.

## State Border Issues

The Albury Base Hospital in New South Wales, which is just across the Victorian New South Wales border, is the major trauma receiving hospital for the far northeast area of Victoria. The Albury Base Hospital, does not submit patient data to any of the DHS or VSTORM datasets. The outcomes for some patients from this area are therefore not available.

## Matching Process

### Victorian Department of Human Services Datasets

Due to privacy concerns with patient data, the data matching process was undertaken by DHS staff only. The data matching process involved multiple steps. First, the EMS data was linked with the VEMD. The initial matching process involved using the date of incident, EMS case number, hospital campus code, and patient date of birth or approximate age (if the date of birth was missing). Second, the EMS data was matched with the VAED utilising linkage data from the VEMD link or just the EMS data. The matching process involved deterministic matching first, then probabilistic matching, for both hospital datasets.

A test dataset of 97 randomly selected mechanism of injury only records was used for a trial linkage with the VEMD. DHS staff found this dataset too small to work with so a larger test dataset consisting of 1,000 randomly selected records from both the sudden deterioration and mechanism of injury only datasets was compiled. When this larger test dataset was used for linking it was found that approximately 50% of the EMS data could be successfully linked to the VEMD using date of incident and EMS case number. The linkage rate improved to 60% when date of birth and hospital name was used.

The EMS trauma dataset that was used for the first real linkage consisted of 6,261 individual records and was linked to both the VEMD and VAED. The EMS dataset consisted of a random sample of 3,200 records from the trauma profile dataset, 1,561 records from the mechanism of injury only dataset and 1,500 records from the sudden deterioration dataset.

### Victorian State Trauma Outcome Registry and Monitoring Dataset

All data files (EMS and VSTORM) were checked to ensure there was only one record for each patient. Both files were then blocked using the date of incident, case number, gender, and age.

The unique variables in both datasets (EMS and VSTORM) for deterministic matching were the date of incident, case number, gender, date of birth, mode of transport (air or road), and EMS (MAS or RAV). The matching was undertaken in Microsoft Access™ using a user-defined query and check with a user-defined function created with visual basic code by the author.

There were two test linkages performed between the EMS and VSTORM datasets. The first matching process, using deterministic matching, was undertaken using 97 mechanism of injury only cases and yielded only one successful link (1%). The second matching process, using deterministic matching, involved a test dataset containing 130 records that were prehospital defined potential major trauma (see Figure 1 for the criteria) either by physiological status, significant pattern of injury or both. This second matching process yielded 30 successful links (23%). This result did not appear correct given the dataset contained records of patients with severe traumatic injuries.

The first real link involved deterministic matching between the EMS (n = 6,261) and VSTORM datasets. The dataset was the same as the one used for the DHS linking. The matching process did not include 897 records (14.3%) from the EMS dataset as they did not contain an EMS case number or patient date of birth.

Given the problems with deterministic matching using the first real EMS and VSTORM datasets, it was considered necessary to undertake probabilistic matching using the same blocking variables (date of incident, case number, gender, and age) for each of the three EMS datasets and the VSTORM dataset. The variables in the trauma profile dataset used for probabilistic matching were date of incident, EMS, case number, gender and age. The variables in the mechanism of injury only and sudden deterioration datasets were date of incident, EMS, case number, gender, age, date of birth, incident location and hospital. The variables in the VSTORM dataset were date of incident, EMS, case number, gender, age, date of birth, incident location postcode and hospital. The results of the matching table were reviewed manually to ensure accuracy of the matching process with the results also compared with the manual matching results.

Finally the files were matched manually to ensure the best possible link. This involved checking a list of all major trauma patients from VSTORM (n = 1,096) against the three EMS datasets. This process was not without its problems. There were some records on the EMS list which could not be confidently matched with the VSTORM list. This inability to confidently link data was due to a lack of PCR data, e.g. case number, approximate patient age, patient date of birth, patient gender, hospital, and incident location postcode.

We found it difficult to cross check if any EMS PCRs were missing from the main VSTORM dataset as the fields used to designate the PCR status had not been routinely completed. According to VSTORM, 80% of the records for 2002 did not have any EMS PCR status recorded.

## Results

### DHS Datasets

In the EMS dataset (n = 6,261) there were 669 (10.7%) records that could not be linked to the VEMD due to the hospital not reporting to the VEMD. There were 66.7% of patients from the EMS dataset located in the VEMD, 28% of these patients were admitted to a hospital ward. When matching EMS data with the VAED, 96% of patients located in the VAED were defined in the VEMD as being admitted to hospital, with 3.7% of patients located in the VAED not found in the VEMD due to the hospital not reporting to the VEMD.

Following the linking process, DHS sent a file that contained the ICD10 diagnosis and procedure codes for the patients that were successfully linked. We were not able to calculate an ISS for the DHS linked data as there was no software available nationally or internationally that was able to calculate the ISS using the ICD10 codes.

### VSTORM Dataset

The first real matching using date of incident and EMS case number yielded 180 (2.9%) matches, this result appear low. When staff from VSTORM and the author reviewed the matching process and undertook different combinations of deterministic matching another 147 records were identified. During this process it was noted that the VSTORM EMS case number field contained some inaccurate data representations. This was reviewed and further cleaned, leading spaces removed and additional text removed, prior to additional matching.

When matching the trauma profile dataset and VSTORM there was a 146% increase in successful matches from the deterministic to the manual matching. There was a 38.8% increase in matches from the probabilistic to the manual matching. However, when reviewing the results of probabilistic matching and comparing it to the results from the manual match, there were only 298 (57.5%) correct matches and 227 false positive matches. See Table 3 for matching figures.

**Table 3:** Matching success for each matching process – trauma profile dataset

	**Trauma Profile Dataset n = 27,600**
	
**Matching Process**	**n**
Deterministic	292
Probabilistic	518
Manual	719

When matching the mechanism of injury only dataset and VSTORM there was a 221% increase in successful matches from the deterministic to the manual matching. There was a 44% decrease in matches from the probabilistic to the manual matching. However, when reviewing the results of probabilistic matching and comparing it to the results from the manual match, there were only 31 (38.3%) correct matches and 50 false positive matches. See Table 4 for matching figures.

**Table 4:** Matching success for each matching process – mechanism of injury only dataset

	**MOI Only Dataset n = 4,571**
	
**Matching Process**	**n**
Deterministic	14
Probabilistic	81
Manual	45

When matching the sudden deterioration dataset and VSTORM there was a 46% increase in successful matches from the deterministic to the manual matching. There was a 10.1% increase in matches from the probabilistic to the manual matching. However, when reviewing the results of probabilistic matching and comparing it to the results from the manual match, there were only 176 (84.6%) correct matches and 35 false positive matches. See Table 5 for matching figures.

**Table 5:** Matching success for each matching process – sudden deterioration dataset

	**Sudden Deterioration Dataset n = 2,687**
	
**Matching Process**	**n**
Deterministic	157
Probabilistic	208
Manual	229

## Discussion

This was the first time in Victoria that a large EMS dataset has been linked to the state hospital datasets, it was also the first time that a large EMS trauma dataset has been linked to the VSTORM dataset. Findings from the linkages have provided a Victorian perspective to patient outcomes from trauma over a twelve month period.

When using the trauma profile EMS dataset only 65.6% of the VSTORM records could be matched. Other EMS linkage studies have had reasonable success with matching EMS and other health related datasets, from 23.9% to 96.1%.[5,14,6,4,17] The majority of these studies used either a combination of deterministic and probabilistic or just probabilistic matching. The international EMS studies did not perform manual matching like in this study so there true linkage rates may have been higher, or lower, depending on the accuracy of the probabilistic matching.

Two of the international EMS linkage studies demonstrated an increase, varying between 17.2% and 66.2% in successful linkages when using probabilistic after deterministic matching.[15,17] Likewise, this study demonstrated an increase from deterministic to probabilistic matching of 32.5% to 478%, and in probabilistic to manual matching of 10.1% to 38.8%. This study reaffirmed that manual matching is the "gold standard" for matching data, however, it is still inappropriate for large population datasets. This study has also established that probabilistic matching may not always produce an accurate link, with the correct probabilistic linkage in this study varying between 38.3% and 84.6%.

Missing and inaccurate data created issues during the matching process, for example the approximate age. An approximate age from the PCR was used when a date of birth (DOB) was not available and in some cases was found to be up to ten years from the actual age. This would have been classed as a miss when using approximate age as one of the matching variables. This inaccuracy in age estimation has not previously been studied in the practicing international paramedic population, however, a study using undergraduate paramedic students by Boyle and Williams highlighted that this cohort was poor at estimating a variety of people's ages.[18]

Paramedics often ask a patient's age in their initial assessment and during transport ask for the complete DOB, it would appear that mathematical errors were made with the year calculation. During the manual matching process inaccuracies were noted in the DOB, mainly the year. It would appear that the patient gave the paramedic their age date and month and the paramedic worked out the birth year from the age. There were many occurrences in the VSTORM dataset where the date and month were correct but the year was different to the EMS dataset by one to three years. Brice et al and Clark et al also specifically commented on missing and inaccurate data, especially the DOB. [5,14] DOB is often one of the main variables used in the matching process.

The missing and inaccurate data, especially case number, gender, date of incident, age or DOB, have affected the electronic, and to a lesser extent, the manual matching process. With the introduction of an electronic PCR in Victoria the incidence of missing and inaccurate data, for example, date of incident, case number, gender, DOB or age, should decrease as these are compulsory fields in the electronic PCR program. If a DOB is entered then the age is calculated by the PCR program, allowing the paramedic to check the correct figures have been entered. Further linkages of Victorian EMS and VSTORM data should be undertaken to aid in the auditing of prehospital trauma management and further the improvement in the matching and successful linkage of the two datasets.

Even though the matching of the EMS dataset to the DHS datasets appeared to be successful we were not able to generate any useful information for the Victorian Prehospital Trauma Triage Study. We were unable to use these results to confirm patient numbers in VSTORM as the data received back from DHS was totally de-identified with no study identifiers. The data received back from DHS contained predominately ICD diagnosis and procedure codes. The ISS is able to be calculated from ICD9 codes using the ICDMap software, however, all hospitals in 2002, coded patient diagnosis and procedures using the ICD10 codes. The mapping of the ICD10 codes back to ICD9 for the calculation of an ISS was not attempted as advice from several sources suggested that the accuracy of the results could not be guaranteed, especially for determining severity of specific injury types. We were able to confirm if a patient had one of the specific types of surgery, however, we could not determine if the surgery was within the first 24 hours of admission, a requirement for hospital defined major trauma.

As part of an ongoing clinical audit process all EMS within Australia should undertake linking of their data with hospital datasets and state registries, i.e. trauma and cardiac arrest, to determine patient outcomes. The determination of patient outcomes will assist in the review of clinical practice guidelines and overall patient management as part of an overall patient safety strategy.

This study is potentially limited as some of the patients who had a fall from standing and did not suffer any physiological distress or have a significant pattern of injury as defined in the Review of Trauma and Emergency Services in Victoria (ROETS) report [19], see Figure 1, may have only been counted as having a traumatic incident and did not have their data included in the study database for further analysis. Some of these patents may have a record in VSTORM even though they had no obvious injury when transported by the EMS. Another organisation was undertaking a study into the outcomes of people who had a fall from standing at about the same time therefore we decided not include this group for additional analysis but just to count the number of incidents, these numbers have been reported elsewhere. [11] Therefore, if all trauma incidents were included in the EMS dataset the linkage results may have been higher. Finally, we were not able to use results from the DHS linkage to confirming the number of patients with hospital defined major trauma.

## Conclusion

This study has demonstrated that EMS data can be successfully linked to other health related datasets using deterministic and probabilistic matching with varying levels of success. Manual matching of datasets is still the gold standard for accurately matching the two datasets, however, large datasets make this process inefficient. The quality of EMS data needs to be improved to ensure better linkage success rates with other health related datasets. This project has provided a platform for national and international comparison to Victorian EMS dataset linkage and trauma outcome studies. This study has also highlighted data linkage issues between EMS and health related datasets relevant to policy makers and researchers.

## Competing interests

The author declares that they have no competing interests.

## Pre-publication history

The pre-publication history for this paper can be accessed here:


